# Pasture species selection for free-range egg production systems: Implications for laying performance, egg quality, hen welfare, and soil functioning

**DOI:** 10.1016/j.psj.2026.107269

**Published:** 2026-06-10

**Authors:** Mónica Gandarillas, Ignacio F. López, José Dörner, Tamara Tadich, Oscar Balocchi, Catalina Guarda

**Affiliations:** a Instituto de Producción Animal. Facultad de Ciencias Agrarias y Alimentarias. Universidad Austral de Chile. Independencia 631. Valdivia. Chile 5110566; b School of Agriculture and Environment, Massey University, Palmerston North, New Zealand; c Instituto de Ingeniería Agraria y Suelos. Facultad de Ciencias Agrarias y Alimentarias. Universidad Austral de Chile; d Centro de Investigación en Suelos Volcánicos, Universidad Austral de Chile, Valdivia, Chile; e Soil and Ecosystem Functions Research Center of Chile (CISFECh), Universidad Austral de Chile, Valdivia, Chile; f Instituto de Ciencia Animal. Facultad de Ciencias Veterinarias. Universidad Austral de Chile, Chile

**Keywords:** Free-range production, Forage selection, Yolk quality, Hen welfare, Soil quality

## Abstract

Free-range egg production systems require pasture management strategies that support hen welfare, egg quality, and environmental sustainability. This narrative review evaluates pasture species selection as a functional component of free-range laying systems, using a bibliographic database compiled and updated by the authors and complemented with targeted searches in scientific databases. The review integrates poultry-specific evidence with supporting information from pasture agronomy, animal behavior, egg-quality research, and soil science. Particular attention is given to temperate species relevant to humid temperate conditions, including *Lolium perenne, Bromus valdivianus, Dactylis glomerata, Trifolium repens, Trifolium pratense, Medicago sativa, Plantago lanceolata, and Cichorium intybus*. Current evidence indicates that pasture intake can improve yolk pigmentation, antioxidant transfer, and omega-3 fatty acid enrichment, although responses vary with botanical composition, pasture availability, season, genotype, and basal diet. Legumes and chicory appear most promising for nutritional contribution and egg-quality responses, whereas grasses are more relevant for persistence, regrowth, and soil cover. Pasture access also supports foraging and exploration, but welfare outcomes depend on vegetation cover, range design, genotype, weather, and substrate condition. From an environmental perspective, hens contribute to nutrient cycling through manure deposition, but uneven range use can create nutrient hotspots, soil compaction, pasture degradation, and risks of nutrient loss. Direct poultry-specific evidence remains limited for several species, especially *B. valdivianus* and *D. glomerata*, whose potential suitability is currently based mainly on agronomic inference. Overall, no single pasture species satisfies all productive, welfare, and soil-function objectives. Multi-species pastures that combine legumes, persistent grasses, and selected broadleaf species may offer the most balanced strategy, but long-term comparative studies are still needed under commercial free-range conditions.

## Graphical abstract

**Figure:**
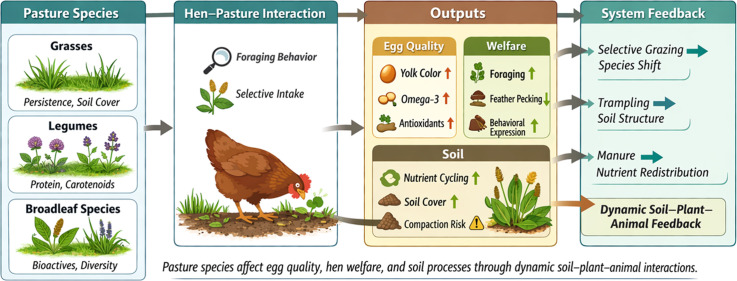

## Introduction

Free-range egg production systems are expanding as markets and regulatory frameworks increasingly prioritize animal welfare and sustainability outcomes (Norwood and Lusk, 2011; Berkhoff et al., 2020). Although knowledge about egg production systems and willingness to pay more for cage-free eggs varies across countries, most consumers still report that hen welfare is an important concern (Berkhoff et al., 2020; Sinclair et al., 2022). In free-range systems, pasture represents more than simply outdoor space: it influences hen behavior and welfare, provides functionally distinct nutrients and bioactive compounds, and contributes to soil and vegetation dynamics within the range area. In Europe, policy changes promoting the transition away from conventional cages toward housing systems with outdoor access have further positioned pasture management as a critical production component rather than an optional design feature (Council Directive 1999/74/EC).

Housing systems strongly influence productive performance, welfare indicators, and egg quality (Wang et al., 2009; Rakonjac et al., 2014a, 2014b). Compared with conventional systems, free-range systems allow hens to express highly motivated natural behaviors such as foraging, dust bathing, and exploration (Lay et al., 2011; Mench and Blatchford, 2014), which are important indicators of positive welfare.

Despite these welfare benefits, free-range systems also present important management challenges. Pasture degradation, parasite exposure, nutrient accumulation in outdoor areas, and uneven paddock utilization are recurrent constraints under commercial conditions (Sosnowka-Czajka et al., 2010; Ricke and Rothrock, 2020). Hens frequently concentrate their activity near poultry houses, resulting in localized vegetation loss, manure accumulation, and deterioration of soil physical properties. These factors can also raise behavioral and health issues in cage-free and organic egg production systems (Bonnefous et al., 2022).

Pasture is therefore not only a welfare resource but also a nutritional and ecological component of free-range systems. Grazed vegetation may contribute nutrients, pigments, antioxidants, and fatty acid precursors that affect egg quality, while simultaneously influencing hen behavior and soil functioning (Horsted et al., 2007a; Karsten et al., 2010; Anderson, 2011; Mugnai et al., 2014).

However, most available research has focused on comparing systems with and without pasture access, rather than evaluating the functional characteristics of pasture vegetation itself or the comparative suitability of different pasture species under poultry grazing. Studies using crop content analysis have demonstrated that free-range hens actively consume herbage and that pasture can contribute nutrients and metabolizable energy to the diet (Horsted et al., 2007a, Horsted et al., 2007b). Moreover, pasture access has been associated with improvements in egg nutritional quality, including higher concentrations of carotenoids, antioxidants, and omega-3 fatty acids (Karsten et al., 2010; Anderson, 2011). Seasonal variation in egg nutrient composition has also been reported in pasture-based systems, reflecting changes in forage availability and environmental conditions throughout the grazing period (Van Duinen et al., 2025). Despite these findings, differences among pasture species—such as grasses, legumes, and broadleaf plants—in palatability, regrowth capacity, nutritional composition, and persistence under poultry grazing remain poorly understood.

This knowledge gap is particularly relevant under humid temperate conditions, where high rainfall, wet soils, rapid seasonal pasture growth, and soil vulnerability add complexity to pasture management and species selection.

Humid temperate systems require specific pasture selection strategies because frequent rainfall can increase soil compaction, mud formation, vegetation loss, and nutrient leaching, particularly near poultry houses and high-use areas. At the same time, rapid pasture growth may provide high-quality forage when species are well adapted and appropriately managed. Findings from drier Mediterranean, arid, or tropical environments may therefore not be directly transferable, because pasture species, growth patterns, soil moisture dynamics, and degradation risks differ among climatic regions.

Pasture in free-range laying systems should therefore be considered a functional soil-plant-hen component rather than only an outdoor area. Through grazing, scratching, trampling, and manure deposition, hens modify vegetation and soil properties, while pasture species influence nutrient intake, egg composition, environmental enrichment, and paddock resilience.

This review aims to integrate current evidence on pasture intake, egg quality responses, welfare implications, soil–plant–hen interactions, and the functional suitability of temperate pasture species, with particular emphasis on humid temperate production systems.

## Review approach and literature selection

This article was conducted as a narrative review. The review was developed from a bibliographic database compiled and updated by the authors since 2020 through previous research projects, grant proposals, and publications related to pasture-based poultry systems, pasture agronomy, soil physical quality, egg quality, and animal welfare. This database included research and review articles, technical publications, and methodological references relevant to free-range laying hens, forage intake, pasture species, soil-plant interactions, and humid temperate pasture systems.

For the present review, this database was re-examined and complemented with targeted searches using Scopus, Web of Science, ScienceDirect, PubMed, and Google Scholar. Searches combined terms related to poultry production and pasture systems, including "free-range laying hens", "pasture intake", "forage selection", "pasture species", "egg quality", "yolk carotenoids", "omega-3 fatty acids", "hen welfare", "soil compaction", "nutrient cycling", and "soil quality".

Studies were prioritized when they addressed laying hens with outdoor or pasture access, pasture intake or botanical composition, egg-quality responses, welfare-related outcomes, or soil and vegetation effects in poultry systems. Because direct evidence on individual pasture species for laying hens is limited, additional studies from pasture agronomy, ruminant grazing systems, plant functional ecology, and soil science were included when they provided relevant information on nutritional composition, regrowth capacity, persistence, root traits, or soil protection. In these cases, the evidence is discussed as indirect and hypothesis-generating rather than as poultry-validated evidence.

**Fig. 1:**
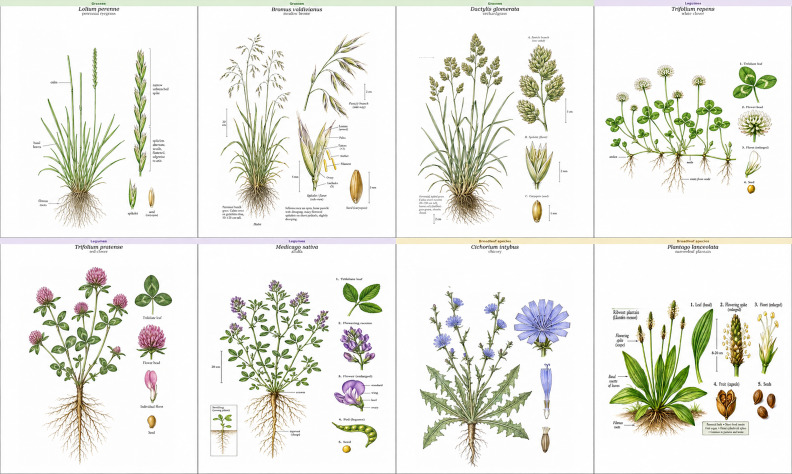
Main temperate pasture species considered for free-range laying hen systems, including grasses, legumes, and broadleaf species with potential roles in forage intake, egg quality, welfare, pasture persistence, and soil functioning.

The pasture species emphasized in this review were selected because of their relevance to humid temperate pasture systems and their potential functional roles in free-range egg production. These species included *Lolium perenne, Bromus valdivianus, Dactylis glomerata, Trifolium repens, Trifolium pratense, Medicago sativa, Plantago lanceolata,* and *Cichorium intybus*. Therefore, this review does not aim to provide an exhaustive global inventory of forage species, but rather a critical synthesis of species with potential relevance for humid temperate free-range laying systems. The main species discussed are shown in Figure 1.

## Pasture intake and nutritional contribution in free-range laying hens

Corn and soybean meal remain the basis of commercial feeding programs for laying hens in both conventional and alternative production systems because they provide standardized concentrations of energy, crude protein, essential amino acids, and minerals required to sustain egg production (NRC, 1994; Rostagno et al., 2017). Under practical feeding conditions, laying hens generally require approximately 15.0-18.0% crude protein, metabolizable energy between 2.55 and 2.86 Mcal/kg, lysine concentrations from 0.69-1.05%, methionine between 0.25-0.45%, and calcium levels above 3.0%, depending on age, genotype, and production stage (NRC, 1994; Rostagno et al., 2017). Daily concentrate intake typically ranges from 100 to 130 g/hen/day, varying with genotype, body weight, egg mass output, and environmental conditions.

In free-range systems, hens consume pasture in addition to concentrate feed, and this supplementary intake may provide nutrients, pigments, vitamins, and bioactive compounds not fully supplied by conventional diets (Horsted et al., 2007a; Singh and Cowieson, 2013). However, quantifying pasture intake remains difficult because grazing behavior varies according to season, pasture availability, botanical composition, climatic conditions, hierarchy, and genotype. Reported estimates differ widely among studies. Hughes and Dun (1983) estimated pasture dry matter intake between 30 and 40 g/hen/day, whereas Antell and Ciszuk (2006) reported similar values under organic systems with continuous pasture access. In contrast, Mugnai et al. (2014) observed fresh pasture intake ranging from 5.7 to 57.9 g/hen/day depending on season, with highest values during spring and lowest during summer, while concentrate intake remained relatively stable between 100 and 116 g/hen/day. Moreover, Briden et al. (2023) observed that hierarchy affected foraging behavior in free-range hens, with more dominant birds using the feeder significantly more than subordinate ones, whereas subordinate birds spent more time foraging on pasture.

In this review, pasture intake refers to the quantity of herbage consumed by hens, forage selectivity refers to the preferential consumption of particular species, plant parts, or phenological stages, and nutrient contribution refers to the digestible and metabolically available nutrients derived from that intake. These concepts are related but not equivalent. A species may be highly selected by hens but contribute modestly to nutrient supply if intake is low, digestibility is limited, or nutrient concentrations decline with maturity.

Variation in pasture intake is partly explained by pasture phenological stage and nutrient composition. Younger vegetative forage generally contains higher water-soluble carbohydrates, lower structural fiber, and greater crude protein concentrations, increasing palatability and facilitating intake (Sossidou et al., 2015). Leaf softness, leaf-to-stem ratio, and botanical composition also influence preference, with hens frequently selecting legumes and broadleaf species when mixed swards are available (Horsted et al., 2007b; Gandarillas et al., 2025).

Legume species such as *Trifolium repens* and *Trifolium pratense* contain high crude protein concentrations and may contribute nutritionally relevant amino acids under grazing conditions. Likewise, broadleaf species such as *Cichorium intybus* and *Plantago lanceolata* provide moderate to high protein concentrations depending on growth stage and environmental conditions (Reverter et al., 1999; Murai et al., 1995; Alomar et al., 2018). However, their quantitative contribution to amino acid supply remains difficult to estimate under field conditions.

Despite this nutritional potential, poultry are traditionally considered limited in their ability to utilize fibrous feedstuffs because of rapid digesta passage rate and relatively low digestive tract capacity compared with herbivorous species. Nevertheless, cecal fermentation may generate metabolically relevant short-chain fatty acids from resistant carbohydrates reaching the hindgut, including acetate, propionate, and butyrate (Svihus, 2014). These fermentation products may contribute modestly to energy metabolism, although direct evidence under free-range conditions remains limited (Ricke et al., 2020).

Pasture intake may also affect concentrate consumption through substitution effects, although results are inconsistent across systems. Oke et al. (2016) observed reduced concentrate intake in layers with access to legume pasture without compromising egg production or body weight, whereas other studies indicate highly variable responses depending on pasture quality, stocking density, and accessibility.

In addition to vegetation, hens may consume insects, seeds, earthworms, grit, and soil particles during outdoor foraging (Horsted et al., 2007a), which further complicates precise estimation of pasture-derived nutrient intake. Crop content analyses also suggest that hens receiving nutritionally less balanced diets actively increase outdoor foraging, indicating that grazing behavior may respond to nutritional motivation. Recent evidence indicates that genotype also influences pasture utilization. Gandarillas et al. (2025) observed greater pasture intake in Isa Brown hens than in White Leghorn hens when expressed relative to live weight, suggesting that body size and genotype-related feeding differences may affect nutrient acquisition from pasture.

Reported pasture intake values are difficult to compare because estimation methods measure different processes. Pasture disappearance methods may overestimate intake because herbage losses can result from trampling, scratching, senescence, or wastage. Crop content analysis provides more direct information on recently ingested material, but it represents a short time window and may be affected by sampling time and individual variation. Microhistological analysis can identify botanical composition of ingested material but is less precise for quantifying total intake. Behavioral observations are useful for evaluating foraging motivation and range use, but they should not be interpreted as direct measures of nutrient contribution.

Overall, pasture contributes nutritionally relevant compounds to free-range laying hens; however, the magnitude and consistency of this contribution depend strongly on pasture species, seasonal conditions, and individual foraging behavior, highlighting the need for improved methodologies to quantify intake and selectivity.

## Effects of pasture intake on egg quality in free-range laying systems

One of the most consistently reported effects of pasture access in free-range laying systems is the modification of egg quality traits, particularly yolk pigmentation, fatty acid profile, and the concentration of antioxidant compounds transferred from feed to the egg (Van Duinen et al., 2025; Mugnai et al., 2014; Karsten et al., 2010). These responses are largely attributed to the ingestion of fresh herbage, which contains carotenoids, polyunsaturated fatty acids, vitamins, and secondary metabolites generally absent or present at lower concentrations in conventional concentrate diets.

Yolk color is one of the most visually apparent egg quality traits affected by pasture intake and remains highly valued by consumers because it is commonly associated with freshness, natural production, and nutritional quality (Berkhoff et al., 2020). In commercial poultry systems, yolk pigmentation is often achieved through synthetic pigments or ingredients rich in xanthophylls, such as corn gluten meal or marigold extracts (Titcomb et al., 2019). In free-range systems, however, hens obtain natural pigments directly from forage, particularly carotenoids such as lutein, zeaxanthin, and β-carotene, which are efficiently absorbed and deposited into the yolk (Skrivan et al., 2015).

The concentration of carotenoids in pasture varies considerably with botanical composition and season. Mugnai et al. (2014) reported that carotenoid concentrations in pasture exceeded those of concentrate diets by approximately eight-fold during spring, 6.9-fold in winter, 3.5-fold in autumn, and 2.8-fold in summer, indicating that seasonal growth conditions strongly influence pigment supply. Consequently, yolk pigmentation responses are often more pronounced during periods of active pasture growth, when leaf tissue is abundant and photosynthetically active.

Legume species such as *Trifolium repens* and *Trifolium pratense* may contribute substantially to pigment intake because of their high carotenoid concentration and digestibility (Reverter et al., 1999; Sossidou et al., 2015), whereas broadleaf species such as *Cichorium intybus* have also shown strong pigmentation potential. Horsted et al. (2007a) reported darker and redder yolks in hens grazing chicory compared with birds grazing grass–clover mixtures, suggesting that botanical composition influences yolk color intensity.

Pasture intake also modifies egg lipid composition, particularly through increased deposition of omega-3 fatty acids in the yolk. Green forage contains α-linolenic acid (C18:3ω3), a precursor of long-chain omega-3 fatty acids synthesized by the hen and transferred into the egg (Lewis et al., 2000). Karsten et al. (2010) found that eggs from hens with pasture access contained higher concentrations of α-linolenic acid than eggs from caged hens, while Mugnai et al. (2014) reported approximately 2.5-fold higher α-linolenic acid and five-fold higher docosahexaenoic acid (DHA, C22:6ω3) in yolks from pastured hens compared with indoor systems.

This enrichment is nutritionally relevant because DHA is associated with cardiovascular and neurological benefits in human nutrition. Moreover, pasture-based enrichment offers a natural alternative to dietary supplementation with fish oil, algae, or flaxseed products commonly used in functional egg production (González-Esquerra and Leeson, 2001). However, the magnitude of enrichment depends strongly on pasture intake level, botanical composition, and the fatty acid profile of the basal concentrate diet.

Fresh forage also contributes antioxidant vitamins that may improve egg nutritional value, particularly through the transfer of tocopherols and carotenoid precursors to the yolk (Karsten et al., 2010; Anderson, 2011). Pasture contains compounds that contribute to vitamin E and vitamin A deposition in eggs, although responses depend on forage composition and basal diet. Seasonal variation in forage quality may further influence antioxidant deposition, as recently observed under pasture-based systems where egg nutrient composition changed throughout the grazing period (Van Duinen et al., 2025).

In addition to carotenoids, fresh pasture may provide tocopherols, polyphenols, and species-specific secondary metabolites that contribute to antioxidant capacity. These compounds may influence egg oxidative stability directly through yolk deposition or indirectly by improving the oxidative status of the hen. However, compared with carotenoids and fatty acids, evidence on the transfer efficiency of pasture-derived polyphenols and other bioactive compounds into eggs remains limited.

In addition to pigments and lipids, pasture intake may also influence internal egg quality traits (Horsted et al., 2007a; Mugnai et al., 2014). Horsted et al. (2007a) observed increased albumen dry matter in eggs from hens grazing chicory compared with grass–clover systems, although evidence regarding albumen height, Haugh units, shell thickness, and shell strength remains inconsistent across studies (Rakonjac et al., 2014a).

Variability among experiments is partly explained by differences in genotype, pasture intake level, concentrate formulation, and climatic conditions (Rakonjac et al., 2014a; Singh and Cowieson, 2013; Küçükyılmaz et al., 2012). In many cases, concentrate diets already contain pigmenting ingredients or fatty acid sources that partially mask pasture effects, while individual differences in pasture use generate substantial within-flock variation (Karsten et al., 2010; Mugnai et al., 2014).

Overall, current evidence indicates that pasture intake improves several egg quality attributes in free-range systems, particularly yolk pigmentation, omega-3 fatty acid concentration, and antioxidant transfer (Anderson, 2011; Karsten et al., 2010). However, the magnitude of these responses depends strongly on pasture species, seasonal growth dynamics, genotype, and basal diet composition (Singh and Cowieson, 2013; Rakonjac et al., 2014a). Therefore, the effects of pasture on egg quality are not determined solely by pasture presence, but by the interaction between pasture species, intake level, seasonal availability, and basal diet composition.

## Welfare implications of pasture access and pasture quality in free-range laying systems

Animal welfare is one of the principal reasons consumers prefer eggs produced under free-range systems, as outdoor access is widely perceived as allowing hens to express natural behaviors that are restricted under conventional housing conditions (Norwood and Lusk, 2011; Berkhoff et al., 2020; Bonnefous et al., 2022). Free-range systems provide opportunities for locomotion, exploration, dust bathing, foraging, wing flapping, and social interactions in a more heterogeneous environment than indoor systems, which improve behavioral expression and are associated with indicators of positive welfare (Lay et al., 2011; Campbell et al., 2020; Calderón-Amor et al., 2026).

Among these behaviors, foraging is considered one of the most highly motivated activities in domestic fowl because of its evolutionary importance in resource acquisition. Even when housed in wire-floored cages and trough-fed concentrate diets that fully satisfy nutrient requirements, hens continue to spend substantial time pecking, scratching while feeding, and investigating the ground (Singh and Cowieson, 2013; Weeks and Nicol, 2006). Foraging is considered a highly motivated need in hens. This has been demonstrated through “contrafreeloading”, whereby hens are willing to “work” by performing foraging behavior (exploration, pecking and scratching at the ground) although food is provided freely available in a trough (Höhne et al., 2023). Therefore, access to pasture provides not only more space allowance and nutritional opportunities, but also behavioral enrichment by increasing environmental complexity and reducing monotony.

Pasture vegetation itself strongly influences outdoor use. Areas with dense vegetation cover are generally more attractive to hens than bare soil because vegetation provides visual shelter, thermal comfort, and opportunities for exploratory pecking (Zeltner and Hirt, 2003; Sossidou et al., 2011). Conversely, poorly vegetated areas or exposed bare soil often reduce outdoor occupancy, particularly in fearful birds. This relationship is important because pasture degradation may progressively reduce the welfare benefits expected from free-range systems. Distance from shelter and paddock design also influence outdoor occupancy, as hens preferentially use protected areas when cover is limited (Hegelund et al., 2005).

One of the major welfare advantages associated with pasture access is the reduction of undesirable behaviors such as feather pecking. Feather pecking is considered a multifactorial behavioral disorder associated with frustration, limited foraging opportunities, social stress, and environmental deficiency, and it remains one of the most important welfare problems in laying hen production systems (Grandin and Deesing, 2013; Rodenburg et al., 2013). Birds maintained in environments that induce fear, restrict exploratory behavior or reduce environmental complexity are more likely to redirect pecking toward conspecific feathers, which may escalate to cannibalism and cause serious welfare and productivity losses (Blokhuis et al., 2007, de Haas et al., 2013).

Providing access to pasture may reduce feather pecking because hens spend more time engaged in ground-directed pecking and foraging activity. Vegetation supplies both edible material and continuous exploratory stimuli, reducing the likelihood that pecking behavior is redirected toward flock mates. Several studies have reported lower feather damage in hens with access to well-managed outdoor areas compared with indoor systems, although responses are highly dependent on flock management and outdoor use intensity (Singh and Cowieson, 2013). Pasture intake may also reduce hunger-related frustration when birds consume additional fibrous material that increases gut fill and prolongs feeding activity. Nutritional factors may also contribute to feather pecking behavior. Deficiencies in key amino acids, particularly lysine, have been associated with increased feather pecking, whereas increased foraging activity and dietary diversity may enhance satiety and reduce behavioral frustration. This suggests that pasture species selection may indirectly influence behavioral outcomes through its effects on feeding behavior and nutrient intake. Although pasture contributes limited digestible energy compared with concentrate diets, its physical presence in the digestive tract may affect satiety and reduce feeding-related competition (Horsted et al., 2007a).

However, free-range systems do not automatically guarantee better welfare outcomes. Outdoor access also introduces several risks, including predation, thermal stress, parasite exposure, disease transmission from wild birds such as avian influenza, Salmonella infection and uneven use of available space (Sosnowka-Czajka et al., 2010; Ricke and Rothrock, 2020; Bonnefous et al., 2022). Hens frequently concentrate near the poultry house, creating localized overgrazing and high manure accumulation, while distant areas remain underutilized.

Individual behavioral differences strongly influence pasture use and marked variation in outdoor ranging behavior has been consistently reported under commercial free-range conditions (Campbell et al., 2016; Göransson et al., 2023). Such differences in outdoor activity may influence nutrient intake and egg quality because birds that forage more intensively are likely to consume greater quantities of pasture-derived resources (Horsted et al., 2007a; Singh and Cowieson, 2013). To improve the efficiency of the use of the outdoor area it is important to consider design of the area, weather conditions and stocking density. In addition, intra-flock differences associated with hen personality and experience should be taken into account (Pettersson et al., 2016; Bonnefous et al., 2022).

Genotype also influences behavioral adaptation to free-range systems. Commercial brown hybrids often show greater outdoor use than lighter white genotypes, partly because of differences in body size, social behavior, and fear response. Gandarillas et al. (2025) observed greater pasture intake in Isa Brown hens than in White Leghorns, suggesting that genotype influences both grazing behavior and nutritional exposure under free-range conditions. Nevertheless, a study based on questionnaires and national data bases on organic and free-range farms in Switzerland, France and The Netherlands found that White hens tended to perform better than Brown hens (Leenstra et al., 2012).

Pasture botanical composition may also affect welfare responses. Broadleaf species and legumes often provide more heterogeneous textures and leaf structures than grasses, potentially stimulating exploratory pecking behavior (Oke and Onagbesan, 2024). In addition, some species maintain higher canopy cover and improve visual complexity, which may increase hens' willingness to move further from shelter (Nagle and Glatz, 2012). For example, Breitsameter et al. (2014) evaluated the effect of 15 sward types on the behavior of laying hens (ISA Warren). The authors found a significant effect of sward type on scratching, plant pecking, and total sward-directed pecking (plant and ground pecking together) in the grass swards, and on ground pecking in the forb swards.

Soil conditions interact strongly with welfare outcomes because hens also use the ground for dust bathing and resting behavior. Sossidou et al. (2011) compared composted vegetable substrate with native topsoil and found that hens showed more comfort behaviors such as standing and dust bathing on softer composted substrates, whereas foraging activity was more frequent on topsoil. These results indicate that substrate quality modifies how hens distribute behaviors outdoors.

Dust bathing is another essential welfare-related behavior influenced by pasture and soil conditions. Dust bathing helps maintain plumage condition by removing excess lipids and particles from feathers (Olsson and Keeling, 2005). Under conventional housing systems sham dust bathing may occur. This behavior lacks positive feedback and negatively affects hen welfare, as reflected in poorer plumage condition (Widowski and Duncan, 2000; Scholz et al., 2014). Free-range systems can facilitate dust bathing if dry and friable substrate is available, but wet or muddy conditions may restrict this behavior and reduce welfare benefits. Thus, in free-range systems the dust bathing material requires regular monitoring and maintenance to avoid it becoming wet and reducing the risk of contact dermatitis (Widowski et al., 2013).

Keel bone fractures are an important welfare concern in laying hens, particularly around the onset and peak of lay (Riber et al., 2018; Rufener and Makagon, 2020). Although cage-free and free-range systems allow hens to perform highly motivated behaviors, they may also increase the risk of keel bone fractures because birds can fly, descend from perches, and collide with objects or other hens within the housing system. Keel bone damage has been reported in non-cage systems, including free-range and barn-housed flocks (Wilkins et al., 2004), and aviary design has been identified as an important management factor (Heerkens et al., 2016). Thus, mitigation strategies in free-range systems should consider perch design, spatial complexity, and nutrition supporting musculoskeletal health. For example, Tarlton et al. (2013) reported reduced keel bone breakage rate and severity in free-range hens fed diets supplemented with omega-3 polyunsaturated fatty acids. However, direct evidence linking pasture use itself with reduced keel bone damage remains limited.

Weather also affects pasture use and welfare responses. Rain, wind, low temperatures, and high solar radiation may reduce outdoor use, independently of pasture quality (Nicol et al., 2003). Therefore, the welfare benefits associated with pasture access depend not only on pasture species but also on shelter availability, paddock design, and climatic adaptation. Producers who open pop holes late or close them early because of adverse weather may restrict hens’ motivation to range (Pettersson et al., 2014).

Overall, pasture access improves welfare by increasing opportunities for highly motivated natural behaviors and reducing behavioral disorders associated with environmental restriction. However, these benefits depend strongly on pasture quality, vegetation cover, genotype, personality traits, soil conditions, and paddock management. Consequently, pasture species selection may influence welfare outcomes indirectly by determining how attractive and functional the outdoor environment remains over time.

## Soil–plant–hen interactions in free-range egg production systems

The sustainability of free-range egg production depends not only on bird performance and welfare but also on the capacity of outdoor areas to maintain soil function and vegetation cover under continuous poultry use. In contrast to indoor systems, free-range hens interact directly with soil through trampling, scratching, manure deposition, and selective use of the paddock, generating complex feedback mechanisms among birds, plants, and soil physical and chemical processes (Sossidou et al., 2011; Hoover et al., 2019).

One of the most immediate effects of hens on soil is nutrient deposition through manure. Poultry excreta contain substantial concentrations of nitrogen, phosphorus, potassium, calcium, and organic matter, which may improve soil fertility when deposition is spatially balanced (Hilimire et al., 2013). In integrated crop–animal systems, this nutrient input can reduce fertilizer requirements and contribute to nutrient recycling within the farm.

Hilimire et al. (2013) evaluated soil fertility after two years of poultry grazing combined with crop production and reported significant increases in soil pH, total carbon, total nitrogen, ammonium, nitrate, Olsen phosphorus, exchangeable potassium, cation exchange capacity, and organic matter compared with ungrazed control plots. These improvements were associated with greater plant biomass and crop height in subsequent bean and sunflower crops, suggesting that poultry grazing may contribute positively to soil fertility when managed appropriately.

The increase in soil pH frequently observed in poultry-grazed systems may be partly explained by the high calcium carbonate concentration in laying hen diets, because calcium supplementation for eggshell formation increases calcium excretion through manure (Sommer and Hutchings, 2001). This process may be particularly relevant in acidic soils, where moderate increases in pH can improve nutrient availability and biological activity.

However, nutrient deposition in free-range systems is rarely homogeneous. Hens tend to concentrate their activity near pop holes, shaded areas, feeders, and shelter structures, creating nutrient hotspots close to the poultry house while distant paddock areas receive little manure input (Leenstra et al., 2012; Sossidou et al., 2015, Bestman and Wagenaar, 2003). This spatial concentration may lead to excessive local nitrogen and phosphorus accumulation, increasing risks of nutrient leaching, ammonia volatilization, and environmental contamination.

In addition to chemical effects, hens strongly modify soil physical properties through trampling and scratching behavior. Trampling increases soil compaction, particularly under wet conditions, reducing pore continuity, water infiltration, and gas exchange (Dec et al., 2012). Compacted soil limits root development, reduces pasture regrowth, and may accelerate pasture degradation over time.

Scratching behavior further alters soil surface structure by removing plant residues, exposing mineral soil, and disturbing aggregates. Although scratching contributes to manure incorporation and weed seed exposure, excessive disturbance may increase vulnerability to surface sealing and erosion during rainfall events. Bare soil generated by repeated scratching near high-use areas is particularly susceptible to crust formation and runoff under humid climatic conditions.

The maintenance of vegetation cover is therefore critical to preserve soil physical stability. Pasture protects soil against raindrop impact, reduces runoff velocity, improves infiltration, and stabilizes surface aggregates through root activity. Species capable of rapid regrowth or lateral expansion through tillering or stolons may therefore be especially valuable under poultry grazing.

Legume species such as *Trifolium repens* contribute additional benefits through biological nitrogen fixation, improving soil nitrogen availability independently of manure inputs. Nitrogen fixation may partially compensate for vegetation removal by hens and support pasture persistence under repeated grazing (Sossidou et al., 2011). However, legumes may also be selectively grazed more intensely than grasses because of higher palatability, potentially reducing their long-term persistence if stocking density is excessive.

Soil moisture strongly modifies the impact of hens on paddocks. Under wet conditions, trampling damages soil structure more rapidly, leading to mud formation, reduced hydraulic conductivity, and increased compaction. Mud accumulation has direct consequences for egg hygiene because dirty feet and plumage increase contamination risk when hens return indoors (Sossidou and Elson., 2009). Muddy paddocks also reduce outdoor attractiveness and may limit behavioral use of pasture.

Soil characteristics also influence hen behavior directly. As discussed in Section 5, Sossidou et al. (2011) observed that substrate type modifies behavioral distribution, with composted soil favoring comfort behaviors and topsoil promoting foraging. These results indicate that substrate texture and friability influence how hens distribute behaviors outdoors.

Pasture species selection may indirectly determine soil resilience under poultry grazing. Grasses such as *Lolium perenne* maintain high tiller density and rapid canopy recovery after defoliation, while *Bromus valdivianus* provides larger tillers and leaf blades that may protect soil surface more effectively under moderate grazing pressure (López et al., 2013; Keim et al., 2015). Broadleaf species such as *Plantago lanceolata* and *Cichorium intybus* develop deep root systems that may improve soil porosity and water infiltration, although their persistence under poultry scratching remains insufficiently studied.

In humid temperate systems, maintaining permanent vegetation cover is particularly important because frequent rainfall increases erosion risk when soil is exposed. Rotational use of paddocks is therefore recommended to allow pasture recovery, reduce parasite accumulation, and prevent long-term soil degradation (Sossidou et al., 2015).

Despite growing recognition of soil importance in free-range systems, most poultry studies still focus primarily on bird responses, while soil physical and biological processes remain poorly quantified (Soares et al., 2022). Measurements such as bulk density, penetration resistance, aggregate stability, infiltration rate, and microbial activity are rarely included in free-range poultry research, limiting understanding of long-term system sustainability.

Although the mechanisms described above are well established in soil science and grazing-system research, their magnitude, spatial distribution, and long-term consequences remain insufficiently validated under commercial free-range poultry conditions. This limitation is important because poultry effects are concentrated in the uppermost soil layers and near high-use areas. Future poultry pasture studies should therefore measure soil physical and biological indicators directly rather than inferring them only from other grazing systems.

Overall, free-range hens act simultaneously as nutrient recyclers, soil disturbers, and vegetation modifiers. The balance among these roles depends on management and pasture composition, reinforcing the importance of integrating soil processes into pasture species selection.

## Comparative suitability of pasture species for free-range laying hens under humid temperate conditions

Selecting suitable pasture species for free-range laying systems requires integrating nutritional value, grazing preference, regrowth capacity, persistence under scratching pressure, seasonal productivity, and effects on soil protection. In contrast to ruminant systems, where pasture species have been extensively evaluated according to intake and productive response, poultry systems remain poorly characterized in terms of plant–animal interactions, despite the central role of pasture in free-range management (Bonnefous et al., 2022; Tufarelli et al., 2018; Singh and Cowieson, 2013).

An ideal pasture for laying hens should combine high palatability, high nutritional quality, rapid regrowth after defoliation, tolerance to trampling and scratching, maintenance of soil cover, and the capacity to provide bioactive compounds transferable to eggs. In addition, the pasture should remain productive under seasonal variation and avoid excessive bare soil exposure that compromises welfare and environmental performance.

Because the available evidence differs among species, this review distinguishes direct poultry evidence from indirect agronomic or grazing-system evidence. This distinction is especially important for *Bromus valdivianus* and *Dactylis glomerata*, for which poultry-specific studies are not yet available. Their potential suitability should therefore be interpreted as an agronomic hypothesis requiring validation under free-range laying hen conditions.

To improve the practical interpretation of species suitability, Table 1 summarizes available information on nutritional and functional characteristics of the species discussed. Where values are not available from poultry-specific studies, this limitation is indicated in the table.

**Table 1:** Nutritional and functional composition of pasture species relevant to free-range laying hens.

	Functional group	Crude protein	Fiber / structural traits	Carotenoids / pigments	Fatty acid / omega-3 relevance	Vitamin E / antioxidants	Major bioactive compounds	Evidence basis
*Lolium perenne*	Temperate perennial grass	15–25% DM depending on phenological stage	Fiber increases rapidly with maturity; dense tillering	Moderate; higher in leafy vegetative tissue	Provides α-linolenic acid in green leaf tissue	Tocopherols present in fresh forage	Mainly chlorophyll-associated antioxidants	Poultry evidence indirect; strong agronomic evidence
*Bromus valdivianus*	Temperate perennial grass	17–27% DM depending on phenological stage	Larger tillers and wider leaves than *L. perenne*; good herbage mass	Not well quantified for poultry systems	Potential source of α-linolenic acid as fresh green forage	Limited species-specific evidence	Not well characterized	Indirect agronomic evidence; poultry validation lacking
*Dactylis glomerata*	Temperate perennial grass	13–22% DM depending on phenological stage	Robust tillers, deeper root system, tolerance to repeated defoliation	Not well quantified for poultry systems	Potential source of α-linolenic acid	Limited species-specific evidence	Not well characterized	Indirect agronomic evidence; poultry validation lacking
*Trifolium repens*	Perennial legume	25–30% DM depending on phenological stage	Lower structural fiber than mature grasses; stoloniferous growth	High carotenoid potential	Good source of α-linolenic acid precursors	Contains antioxidant compounds typical of leafy legumes	Phenolics and flavonoid-related compounds	Some poultry evidence for preference/intake; strong agronomic evidence
*Trifolium pratense*	Perennial legume	20–25% DM depending on phenological stage	Erect growth; less tolerant of repeated severe defoliation than white clover	High carotenoid potential	Good source of α-linolenic acid precursors	Contains antioxidant compounds	Isoflavones and phenolic compounds	Mostly indirect for laying hens; strong forage evidence
*Medicago sativa*	Perennial legume	20–25% DM depending on phenological stage	Erect growth; crown sensitive to repeated damage	High carotenoid content	Good source of α-linolenic acid precursors	Rich in tocopherols and antioxidant compounds	Saponins, flavonoids, phenolics	Poultry diet evidence exists; grazing persistence evidence limited
*Plantago lanceolata*	Perennial broadleaf herb	15–20% DM depending on phenological stage	Rosette habit; accessible leaves; fibrous roots	Moderate; less characterized than legumes	Potential green-forage omega-3 contribution	Antioxidant activity reported	Acteoside, aucubin, catalpol	Indirect evidence; poultry-specific transfer data limited
*Cichorium intybus*	Perennial broadleaf herb	20–25% DM depending on phenological stage	Deep taproot; large accessible leaves; crown sensitivity possible	High pigmentation potential reported in poultry studies	Potential source of α-linolenic acid precursors	Contains antioxidant compounds	Condensed tannins, phenolics, sesquiterpene lactones	Poultry evidence available for intake and egg traits; persistence evidence limited

**Note:** Values should be interpreted as approximate or relative because pasture composition varies with cultivar, soil fertility, phenological stage, season, and management. Evidence basis distinguishes direct laying-hen studies from indirect evidence derived from pasture agronomy, ruminant grazing, soil science, or poultry feeding studies. Crude protein values and nutritional characterizations were based mainly on Fraser and Rowarth (1996), Anrique et al. (2014), Reverter et al. (1999), Alomar et al. (2018), and species-specific references discussed in the text. Functional traits and poultry relevance were supported by Horsted et al. (2007a,b), Karsten et al. (2010), Sossidou et al. (2011, 2015), López et al. (2013), Keim et al. (2015), Calvache et al. (2020), and related references cited in Sections 3–7.

### Grasses

Among temperate grasses, *Lolium perenne* is one of the most widely used species in pasture-based livestock systems because of its high digestibility, rapid establishment, and strong tillering capacity (López et al., 2013; Calvache et al., 2020). Its dense tiller population allows rapid canopy recovery after grazing and contributes to maintaining soil cover under repeated defoliation (Calvache et al., 2020; López et al., 2013). These characteristics make it a practical candidate for poultry systems where pasture persistence is challenged by scratching and trampling (Sossidou et al., 2015).

However, although *Lolium perenne* offers good regrowth, its relatively narrow leaves and fine tiller structure may influence accessibility for hens compared with larger-leaved grasses. Poultry grazing differs from ruminant grazing because hens peck selectively at leaf tissue rather than harvesting bulk forage mass. Therefore, leaf accessibility may be particularly relevant for determining practical intake.

*Bromus valdivianus* represents an interesting alternative under humid temperate conditions, with fertile volcanic soils (Keim et al., 2015; Ordóñez et al., 2018). This species produces larger tillers and wider leaves than *Lolium perenne* while maintaining comparable herbage mass production (Ordóñez et al., 2018). Its larger leaf blades may improve accessibility for hens, although this hypothesis has not yet been tested under poultry grazing conditions.

Despite these advantages, *Bromus valdivianus* has not been evaluated directly in poultry systems, and information regarding hen preference, selective defoliation, and persistence under scratching remains unavailable. This represents an important research gap because its morphology suggests potential suitability under poultry grazing conditions.

*Dactylis glomerata* may also represent a useful temperate grass in free-range laying systems because of its tolerance to repeated defoliation, relatively deep root system, and stable productivity under variable moisture conditions (Gatti et al., 2016). Compared with finer-leaved grasses, orchardgrass develops wider leaves and robust tillers, which may improve accessibility for hens under vegetative growth stages, although direct evidence under poultry grazing remains unavailable.

Compared with grasses, hens often consume lower proportions of mature grass tissue when mixed botanical options are available, likely because grasses accumulate structural fiber more rapidly as leaves age (Horsted et al., 2007b). Therefore, grasses may contribute more strongly to soil protection than to nutrient intake unless grazing occurs under vegetative conditions.

### Legumes

Legumes are among the most promising pasture components for free-range laying systems because they combine high crude protein concentration, favorable amino acid profiles, and high concentrations of pigments and essential fatty acid precursors.

*Trifolium repens* is particularly attractive because of its stoloniferous growth habit, which allows rapid lateral expansion, canopy recovery after defoliation, and maintenance of soil cover under repeated grazing (Alarcón et al., 2010; Sossidou et al., 2015). The stolon network contributes strongly to soil coverage and may improve resilience under scratching pressure compared with erect species (Sossidou et al., 2011). In compositional terms, *Trifolium repens* contains approximately 24.1% crude protein, lysine concentrations near 4.5%, and methionine around 1.3% on a dry matter basis (Reverter et al., 1999). Its high palatability also makes it highly attractive to hens when mixed botanical options are available (Horsted et al., 2007b; Gandarillas et al., 2025).

*Trifolium pratense* also offers high nutritional value, with crude protein concentrations near 21.5% and amino acid profiles similar to white clover (Reverter et al., 1999). In addition, red clover provides significant carotenoid concentrations and contributes omega-3 fatty acid precursors that may enhance egg nutritional quality.

However, unlike *Trifolium repens, Trifolium pratense* has an erect growth habit and lower tolerance to repeated severe defoliation, which may reduce persistence under continuous poultry use. Its larger leaves may improve short-term intake, but persistence may be compromised under high stocking density. Legumes also provide agronomic advantages through biological nitrogen fixation, improving soil nitrogen supply and reducing dependence on external nitrogen fertilization (Sossidou et al., 2011). Nevertheless, because hens may preferentially consume legumes, maintaining balanced botanical composition under long-term use may require rotational grazing.

*Medicago sativa* is nutritionally attractive because of its high crude protein concentration, elevated carotenoid content, and recognized contribution to antioxidant transfer in eggs when included in poultry diets or grazing systems (Karsten et al., 2010; Tufarelli et al., 2018). However, its erect growth habit and crown sensitivity may reduce persistence under repeated scratching and localized trampling, particularly under humid conditions (Gatti et al., 2016).

### Broadleaf species

Broadleaf species such as *Plantago lanceolata* and *Cichorium intybus* offer functional advantages that extend beyond basic nutrient supply. *Plantago lanceolata* has moderate crude protein concentration compared with legumes but contains bioactive compounds including acteoside, aucubin, and catalpol, which possess antioxidant and anti-inflammatory activity (Murai et al., 1995). These compounds may influence gut health and oxidative status, although their direct transfer into eggs remains insufficiently studied.

Agronomically, *Plantago lanceolata* develops a rosette growth form and fibrous root system that improves tolerance to dry periods and contributes to soil stabilization (Pol et al., 2021). Its leaf architecture may also facilitate selective pecking by hens because leaves remain accessible near soil level.

*Cichorium intybus* has attracted particular interest because of its condensed tannin content and potential antiparasitic properties under grazing systems (Horsted et al., 2007a; Alomar et al., 2018). Condensed tannins may reduce survival of gastrointestinal nematode larvae in grazing systems and could therefore provide indirect health benefits in outdoor poultry systems, although direct evidence in laying hens remains limited (Alomar et al., 2018). Nutritionally, chicory contains crude protein concentrations ranging from 12.7 to 20.0% depending on phenological stage and growing conditions (Alomar et al., 2018). Horsted et al. (2007a) reported pasture intake of 50 to 70 g dry matter per hen per day when chicory was offered, together with improvements in albumen dry matter and yolk redness.

Chicory also produces deep taproots that improve access to water under dry conditions and may contribute to greater soil porosity (Uteau et al., 2013). However, persistence under repeated poultry scratching remains uncertain, because crown damage under repeated defoliation may reduce regrowth when grazing pressure is excessive (Alomar et al., 2018).

### Functional comparison across species

When comparing species, no single pasture species satisfies all criteria simultaneously. Grasses provide superior soil protection and regrowth capacity, legumes provide superior nutritional value and egg enrichment potential, and broadleaf species contribute functional compounds and seasonal resilience. A functional comparison of key pasture species highlights the trade-offs among nutritional value, persistence, and ecological function, reinforcing that no single species fulfills all requirements simultaneously (Table 2).

**Table 2:** Functional suitability and evidence strength of pasture species for free-range laying systems under humid temperate conditions.

Species	Hen preference / intake potential	Egg-quality potential	Welfare / enrichment value	Regrowth and persistence	Soil protection	Main limitation	Evidence strength
*Lolium perenne*	Moderate; best when leafy and vegetative	Moderate; contributes pigments and fatty acid precursors when actively growing	Provides cover and foraging substrate	High tillering and rapid recovery	High because of dense cover	May become less attractive as fiber increases	Moderate: agronomic evidence strong, poultry-specific evidence limited
*Bromus valdivianus*	Potentially moderate to high because of wider leaves	Unknown; possible green-forage contribution	Potentially good cover and structural complexity	Good under humid temperate conditions	Potentially high	No direct poultry studies	Low for poultry; moderate agronomic support
*Dactylis glomerata*	Potentially moderate in vegetative stage	Unknown to moderate	Provides structural cover	Good tolerance to repeated defoliation	Moderate to high because of root system and persistence	No direct poultry studies	Low for poultry; moderate agronomic support
*Trifolium repens*	High; often preferred in mixed swards	High because of protein, carotenoids, and fatty acid precursors	High, because palatable leaves stimulate foraging	Good lateral recovery through stolons	Moderate to high if maintained	May be overgrazed because of high preference	Moderate to high
*Trifolium pratense*	High short-term intake potential	High nutritional and pigment potential	High as palatable forage	Moderate to low under repeated severe defoliation	Moderate	Lower persistence under continuous poultry use	Moderate
*Medicago sativa*	High nutritional potential, but grazing suitability variable	High because of carotenoids and antioxidants	Moderate to high	Moderate to low under scratching and trampling	Moderate because of deep roots, but canopy gaps may occur	Crown sensitivity and persistence risk	Moderate for diet evidence; low for free-range persistence
*Plantago lanceolata*	Potentially high because leaves remain accessible	Moderate; bioactive potential not fully tested in eggs	High structural and sensory diversity	Moderate; drought-tolerant	Moderate to high through root contribution	Limited poultry-specific evidence	Low to moderate
*Cichorium intybus*	High; poultry studies report substantial intake	High; associated with yolk redness and albumen changes	High because of large leaves and foraging stimulation	Moderate; may decline with crown damage	Moderate; deep taproot may improve porosity	Persistence under scratching uncertain	Moderate to high for intake/egg traits; lower for persistence

Evidence strength reflects the availability of poultry-specific evidence, not only agronomic knowledge.

“Low” indicates that suitability is mainly inferred from plant traits or non-poultry grazing studies.

“Moderate” indicates partial poultry evidence or strong indirect support.

“High” would require repeated poultry-specific studies across seasons and management conditions; currently, few species meet this threshold.

Therefore, pasture selection may need to prioritize objectives depending on production context. Systems focused on maximizing egg nutritional quality may favor legumes and chicory, whereas systems prioritizing pasture persistence and soil cover may require greater grass dominance.

At present, one of the largest knowledge gaps is the absence of long-term comparative studies evaluating how hens modify botanical composition over time through selective grazing and scratching. Such information is essential because pasture species that appear highly attractive initially may decline rapidly under continuous poultry use.

Consequently, pasture selection should be approached as a multi-criteria decision that balances nutritional contribution, animal preference, persistence under poultry disturbance, and soil protection, rather than as a purely nutritional choice.

## Knowledge gaps and future research directions

Despite the growing expansion of free-range egg production systems worldwide, several critical and unresolved knowledge gaps remain regarding the integration of pasture species into poultry production. Most existing studies have focused on short-term responses such as egg production, yolk pigmentation, and general behavioral observations, whereas long-term interactions among hens, pasture growth and botanical composition dynamics, and soil processes remain poorly understood.

One of the main limitations in current literature is the lack of standardized methodologies for estimating pasture intake in laying hens. Reported pasture consumption values vary widely among studies because different methods have been used, including indirect disappearance estimates, crop content analysis, and behavioral observations, often without simultaneously quantifying pasture availability or botanical composition (Horsted et al., 2007a; Mugnai et al., 2014). The use of different methods across studies — including indirect disappearance estimates, crop content analysis, and marker-based techniques — limits the comparability of reported intake values.

In addition, little information exists regarding selective grazing at the plant and tissue level under poultry systems. In grazing research, selective defoliation has been studied in detail by evaluating leaf age, tiller structure, and grazing preference across plant strata (Hodgson, 1979; Hodgkinson et al., 2011). Comparable approaches have rarely been applied to poultry systems, despite evidence that hens selectively consume specific botanical groups and plant parts.

Long-term persistence of pasture species under poultry grazing also remains largely unexplored. Many studies evaluate pasture use for only a few weeks or months, which is insufficient to determine how repeated scratching, trampling, and selective defoliation modify botanical composition over time. Species highly preferred by hens may decline rapidly under continuous use, while less preferred species may dominate the pasture progressively, altering both nutritional value and soil cover.

The interaction between genotype and pasture use represents another important research area. Recent evidence suggests that commercial hen genotypes differ in pasture intake, exploratory behavior, and use of outdoor space (Gandarillas et al., 2025; Göransson et al., 2023). However, most pasture studies still treat laying hens as behaviorally homogeneous populations. Understanding genotype × pasture interactions may improve matching of bird type with free-range environments.

Similarly, personality-based variation in outdoor use deserves greater attention. Fearful hens often remain indoors even when high-quality pasture is available, whereas exploratory individuals may obtain greater nutritional and behavioral benefits from outdoor access. These behavioral differences may generate within-flock variability in egg composition, welfare outcomes, and health exposure.

Soil responses are another underdeveloped area of research. Although manure deposition effects on soil fertility have been described, measurements of soil physical quality remain scarce. Variables such as bulk density, aggregate stability, penetration resistance, infiltration rate, and microbial activity are rarely incorporated into poultry grazing studies (e.g., Sossidou et al., 2011; Soares et al., 2022), despite their importance for long-term paddock sustainability.

The difficulty of measuring the effects of poultry grazing in the uppermost soil centimeters could limit the study of their impact on soil physical properties, which may be addressed by adjusting or developing methodologies for this purpose.

Future research should therefore adopt integrated experimental approaches that simultaneously evaluate pasture growth, botanical persistence, hen behavior, nutrient intake, egg quality responses, welfare indicators, and soil properties over extended periods. Such integrated designs are necessary to identify pasture species capable of sustaining both productive and ecological functions under commercial free-range conditions.

Particular attention should also be given to humid temperate systems, where rainfall intensity, soil moisture, and seasonal growth strongly affect pasture persistence and soil vulnerability. Species adapted to these conditions may differ substantially from those recommended under Mediterranean or tropical environments.

Ultimately, future pasture research in free-range poultry should move beyond describing pasture presence and begin evaluating pasture functionality as a biological component of the production system.

## Conclusions

Pasture represents far more than a simple environmental enrichment component in free-range egg production systems. Its botanical composition directly influences nutrient intake, egg quality, hen behavior, and the ecological functioning of outdoor paddocks.

Current evidence indicates that pasture intake contributes carotenoids, omega-3 fatty acid precursors, vitamins, and antioxidants that improve yolk pigmentation and nutritional composition of eggs. At the same time, pasture access promotes highly motivated natural behaviors such as foraging and exploration, contributing to improved welfare when vegetation cover is adequate.

However, no single pasture species satisfies all production objectives simultaneously. Grasses contribute persistence and soil protection, legumes provide superior nutritional value, and broadleaf species offer functional compounds and seasonal resilience. These results highlight inherent trade-offs among nutritional value, grazing tolerance, persistence, and soil function, reinforcing the need for a multi-species approach in free-range systems.

These trade-offs support the use of multi-species pastures that combine persistent grasses for soil cover, legumes for nutritional value and nitrogen fixation, and selected broadleaf herbs for functional diversity. However, this recommendation still requires validation through long-term poultry-specific studies under commercial free-range conditions.

Consequently, pasture species selection should integrate nutritional value, hen preference, persistence under poultry grazing, and soil stability. Moreover, pasture should be understood as part of a dynamic soil–plant–animal system, where species composition influences not only productivity but also behavioral and ecological outcomes over time.

## CRediT authorship contribution statement

**Mónica Gandarillas:** Writing – review & editing, Writing – original draft, Visualization, Validation, Supervision, Resources, Project administration, Methodology, Investigation, Formal analysis, Conceptualization. **Ignacio F. López:** Writing – review & editing, Writing – original draft, Supervision, Formal analysis, Conceptualization. **José Dörner:** Writing – review & editing, Writing – original draft, Investigation, Formal analysis, Conceptualization. **Tamara Tadich:** Writing – review & editing, Writing – original draft, Investigation, Formal analysis, Conceptualization. **Oscar Balocchi:** Writing – review & editing, Writing – original draft, Investigation, Formal analysis, Conceptualization. **Catalina Guarda:** Writing – review & editing, Writing – original draft, Formal analysis, Data curation.

## Disclosures

All authors declare no conflict of interest.
